# Three dimensional Ti_3_C_2_ MXene nanoribbon frameworks with uniform potassiophilic sites for the dendrite-free potassium metal anodes†

**DOI:** 10.1039/d0na00515k

**Published:** 2020-07-27

**Authors:** Haodong Shi, Yanfeng Dong, Shuanghao Zheng, Cong Dong, Zhong-Shuai Wu

**Affiliations:** State Key Laboratory of Catalysis, Dalian Institute of Chemical Physics, Chinese Academy of Sciences 457 Zhongshan Road Dalian 116023 China wuzs@dicp.ac.cn; University of Chinese Academy of Sciences 19 A Yuquan Rd, Shijingshan District Beijing 100049 China; Dalian National Laboratory for Clean Energy, Chinese Academy of Sciences 457 Zhongshan Road Dalian 116023 China; Department of Chemistry, College of Sciences, Northeastern University 3-11 Wenhua Road Shenyang 110819 China

## Abstract

Potassium (K) metal batteries hold great promise as an advanced electrochemical energy storage system because of their high theoretical capacity and cost efficiency. However, the practical application of K metal anodes has been limited by their poor cycling life caused by dendrite growth and large volume changes during the plating/stripping process. Herein, three-dimensional (3D) alkalized Ti_3_C_2_ (a-Ti_3_C_2_) MXene nanoribbon frameworks were demonstrated as advanced scaffolds for dendrite-free K metal anodes. Benefiting from the 3D interconnected porous structure for sufficient K accommodation, improved surface area for low local current density, preintercalated K in expanded interlayer spacing, and abundant functional groups as potassiophilic nuleation sites for uniform K plating/stripping, the as-formed a-Ti_3_C_2_ frameworks successfully suppressed the K dendrites and volume changes at both high capacity and current density. As a result, the a-Ti_3_C_2_ based electrodes exhibited an ultrahigh coulombic efficiency of 99.4% at a current density of 3 mA cm^−2^ with long lifespan up to 300 cycles, and excellent stability for 700 h even at an ultrahigh plating capacity of 10 mA h cm^−2^. When matched with K_2_Ti_4_O_9_ cathodes, the resulting a-Ti_3_C_2_–K//K_2_Ti_4_O_9_ full batteries offered a greatly enhanced rate capacity of 82.9 mA h g^−1^ at 500 mA g^−1^ and an excellent cycling stability with high capacity retention (77.7% after 600 cycles) at 200 mA g^−1^, demonstrative of the great potential of a-Ti_3_C_2_ for advanced K-metal batteries.

## Abstract 2

Three dimensional alkalized Ti_3_C_2_ MXene nanoribbon frameworks are demonstrated as advanced scaffolds for dendrite free potassium metal anodes.

## Graphical abstract

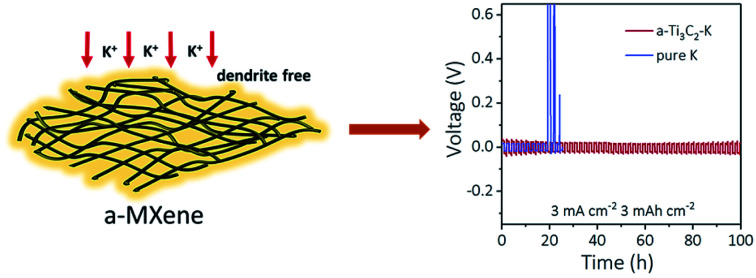

## Introduction

The ever-increasing energy consumption has motivated the rapid development of the renewable and efficient electrochemical energy storage devices for electronics, electric vehicles, and smart grid applications.^1–4^ As one of the most promising rechargeable batteries, K metal batteries have drawn much attention because of their high specific capacity (687 mA h g^−1^), rich natural abundance and a relative low redox potential (−2.92 V *vs.* the standard hydrogen electrode).^5–7^ In addition, when paired with high capacity cathodes, such as sulfur (S) and oxygen (O_2_), the corresponding K–O_2_ and K–S batteries are regarded as the future generation of energy-storage devices.^8–11^ However, many challenges including K dendrites, infinite volumetric expansions and the inactive dead K of the anode result in poor reversibility and safety issues that have substantially hampered their practical commercial applications.^12–14^

To date, considerable efforts have been put in to prevent the dendrite growth, including (i) employing K metal-based alloys (*e.g.* K–Na alloy) with low melting points to eliminate the dendrites;^15,16^ (ii) engineering a solid electrolyte interphase (SEI) with high mechanical properties as a protection layer to suppress dendrite growth;^17^ and (iii) modifying the concentration or optimizing the composition of liquid electrolyte to optimize the SEI layer for guiding uniform K deposition.^18^ However, these strategies are insufficient. For example, manipulation of the liquid state K–Na alloy may be complicated and even cause safety concerns, and solid state electrolytes usually suffer from low ionic conductivity. Meanwhile, electrolyte optimization can't suppress the infinite volumetric changes that occur during cycling, which greatly hampers the development of K metal batteries. In contrast, the introduction of K into three dimensional (3D) robust hosts with high electrical conductivity is expected to simultaneously block the K dendrites by decreasing the local current density and suppress the volume fluctuations of the K metal anode.^19,20^ To date, various 3D porous frameworks have been reported as effective scaffolds for K anodes, including carbon nanotubes and 3D graphene–Cu.^21,22^ However, those reported 3D electrodes lack potassiophilic sites, leading to a relatively uneven K nucleation process. Thus, the introduction of potassiophilic species into 3D conductive frameworks is regarded as one of the most promising ways to produce dendrite-free K metal anodes.

MXenes are a new kind of two-dimensional (2D) carbide or nitride, typically Ti_3_C_2_T_*x*_, where T_*x*_ represents the surface functional groups.^23–26^ MXenes have high electronic conductivity (∼10^4^ S cm^−1^), a fast ion diffusion capability, and inherent surface terminations (F, O, OH, Cl) that have particularly been shown to have strong affinities for alkali metal ions. As a result, MXenes have been widely demonstrated as an appropriate scaffold for dendrite-free Li and Na metal deposition.^27–29^ However, traditional multilayer MXenes possess low porosities for accommodating the large volume variations of K during stripping and plating, and delaminated MXene nanosheets with reduced layers tend to undergo severe aggregation, seriously reducing the exposed active sites for inducing uniform K deposition, which results in low capacity and cycling stability. Therefore, designing 3D MXene networks with sufficient porous structures and enriched potassiophilic sites would greatly facilitate high performance K metal anodes, which has thus far not been achieved.

In this work, we rationally designed and prepared 3D alkalized Ti_3_C_2_ (a-Ti_3_C_2_) nanoribbon frameworks as advanced hosts for dendrite free K deposition *via* electrodeposition. The 3D interconnected macroporous structures inhibited K volume variation, and the abundant preintercalated K ions and functional groups provided potassiophilic sites for uniform K deposition; as a result, the a-Ti_3_C_2_–K anodes realized a flat and homogenous K metal deposition at a high current density and plating capacity. Consequently, a high coulombic efficiency of 99.4% coupled with a long stability of 300 cycles were achieved. Meanwhile, an ultra-low overvoltage of 11 mV and cycling lifespan up to 700 h were obtained in the absence of K dendrites. By pairing a-Ti_3_C_2_–K with K_2_Ti_4_O_9_ (KTO) cathode, the obtained full batteries showcased superior rate performance and cycling stability, which demonstrates the application potential of a-Ti_3_C_2_ frameworks for safe K metal batteries with high energy density.

## Results and discussion

### Fabrication and characterization of a-Ti_3_C_2_ MXene nanoribbon frameworks

**Fig. 1:**
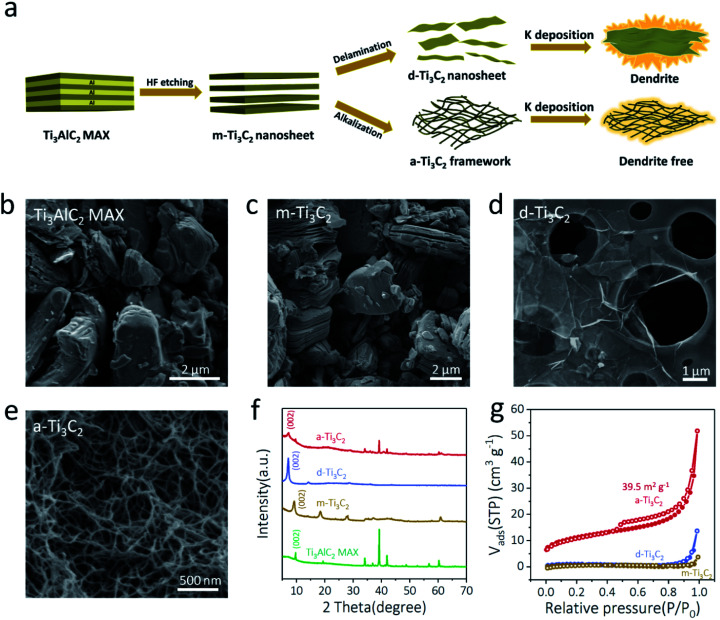
Preparation and characterization of different Ti_3_C_2_ based MXene materials. (a) Schematic of the synthesis of a-Ti_3_C_2_ MXene nanoribbon framework and d-Ti_3_C_2_ MXene nanosheet for K deposition. (b–e) SEM images of (b) Ti_3_AlC_2_ MAX, (c) m-Ti_3_C_2_ MXene nanosheet, (d) d-Ti_3_C_2_ MXene nanosheet and (e) a-Ti_3_C_2_ MXene nanoribbon framework. (f) XRD patterns of Ti_3_AlC_2_ MAX, m-Ti_3_C_2_ MXene nanosheet, d-Ti_3_C_2_ MXene nanosheet and a-Ti_3_C_2_ MXene nanoribbon. (g) Nitrogen sorption isotherms of m-Ti_3_C_2_ MXene nanosheet, d-Ti_3_C_2_ MXene nanosheet and a-Ti_3_C_2_ MXene nanoribbon.

The synthesis of various Ti_3_C_2_ based MXene materials, including multilayer Ti_3_C_2_ (m-Ti_3_C_2_) MXene nanosheets, delaminated Ti_3_C_2_ (d-Ti_3_C_2_) MXene nanosheets, and porous a-Ti_3_C_2_ MXene nanoribbon frameworks for K plating, is illustrated in Fig. 1a. First, the precursor of the Ti_3_AlC_2_ MAX phase with a densely layer-stacked structure (Fig. 1b and S1, ESI†) was obtained *via* ball-milling powders of Ti, Al, and graphite at a molar ratio of 3 : 1.1 : 1.88 at 1550 °C for 2 h in an argon flow atmosphere.^30,31^ Then, accordion-like m-Ti_3_C_2_ MXene with a thickness of 19–52 nm (Fig. 1c and S2, ESI†) was prepared by selectively etching the Al layers of the prepared Ti_3_AlC_2_ MAX in HCl and LiF solution.^32,33^ Third, flat, transparent, and ultrathin 2D d-Ti_3_C_2_ MXene nanosheets (Fig. 1d and S3, ESI†) were successfully obtained by sonication-assisted delamination from the m-Ti_3_C_2_ MXene.^34,35^ Meanwhile, 3D a-Ti_3_C_2_ frameworks with interconnected porous structures (Fig. 1e and S4, ESI†) were easily obtained by shaking the m-Ti_3_C_2_ MXene with KOH solution under an Ar protection atmosphere.^36,37^ As shown in Fig. 1f, X-ray diffraction (XRD) patterns validated the successful fabrication of the various Ti_3_C_2_ MXene based products. Notably, the interlayer spacing of (002) for the a-Ti_3_C_2_ frameworks was 12.2 Å, obviously larger than those of the pure Ti_3_AlC_2_ MAX phase (9.1 Å) and m-Ti_3_C_2_ MXene (9.6 Å) due to K ion intercalation.^36^ In addition, the a-Ti_3_C_2_ MXene nanoribbon with the 3D woven-like frameworks and open macropores exhibited a much improved specific surface area (∼39.5 m^2^ g^−1^) relative to those of d-Ti_3_C_2_ MXene (∼3.6 m^2^ g^−1^) and m-Ti_3_C_2_ MXene (∼2.6 m^2^ g^−1^) (Fig. 1g). It is noteworthy that the 3D interconnected porous structures were beneficial for rapid K ion diffusion and electron transport.^38,39^ Thus, enhanced ionic and electronic conductivity was realized for the a-Ti_3_C_2_ MXene nanoribbon electrodes.

**Fig. 2:**
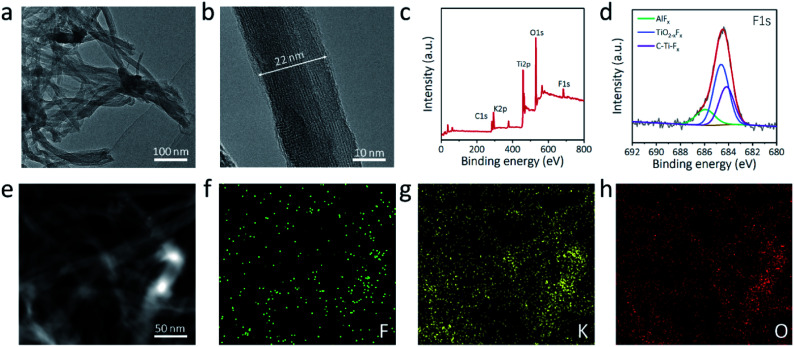
Morphological characterization of a-Ti_3_C_2_ MXene nanoribbon frameworks. (a) TEM image, (b) HRTEM image, (c) XPS full spectrum and (d) high-resolution F1s XPS spectrum of a-Ti_3_C_2_ nanoribbon frameworks. (e) TEM image and (f and g) corresponding EDX elemental mapping images of (f) F, (g) K and (h) O in a-Ti_3_C_2_ nanoribbon frameworks.

Transmission electron microscopy (TEM) was adopted to further examine the morphology of the a-Ti_3_C_2_ MXene nanoribbon. A 3D intertwined microstructure of the elongated a-Ti_3_C_2_ MXene nanoribbon was clearly evident (Fig. 2a). High-resolution TEM (HRTEM) revealed the high phase crystallinity and widths of 10–40 nm (Fig. 2b and S5, ESI†). Moreover, a large interlayer spacing of 1.3 nm, corresponding to the (002) plane of the a-Ti_3_C_2_ MXene nanoribbon, was identified (Fig. S5, ESI†). X-ray photoelectron spectroscopy (XPS) was applied for identifying the elements of the a-Ti_3_C_2_ MXene frameworks. Obvious signals of C1s (290.3 eV), K2p (299.8 eV), Ti2p (468.0 eV), O1s (539.0 eV) and F1s (694.3 eV) were observed for the a-Ti_3_C_2_ MXene nanoribbon (Fig. 2c). Notably, the contained F and O elements are favorable for uniform alkali metal deposition (Fig. 2d and S6, ESI†).^40^ Moreover, energy dispersive X-ray (EDX) element mapping showed the uniform distribution of F, K, and O in the a-Ti_3_C_2_ MXene nanoribbon framework (Fig. 2e and f).

### Electrochemical performance of a-Ti_3_C_2_ hybrid anodes

**Fig. 3:**
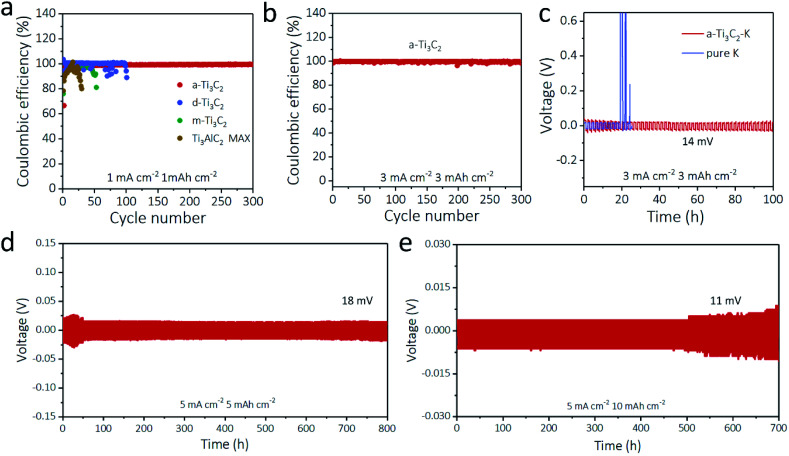
Electrochemical performance of a-Ti_3_C_2_ MXene nanoribbon frameworks for K anodes. (a) Coulombic efficiencies of a-Ti_3_C_2_ MXene, d-Ti_3_C_2_ MXene, m-Ti_3_C_2_ MXene, Ti_3_AlC_2_ MAX and bare Cu electrodes with a K deposition capacity of 1 mA h cm^−2^ at 1 mA cm^−2^. (b) Coulombic efficiency of a-Ti_3_C_2_ MXene electrode at a high current density of 3 mA cm^−2^ for 3 mA h cm^−2^. (c) Galvanostatic cycling of symmetric batteries based on a-Ti_3_C_2_–K and pure K electrodes at 3 mA cm^−2^ for 3 mA h cm^−2^. (d and e) Long-life symmetric batteries galvanostatic cycling of a-Ti_3_C_2_–K electrodes at 5 mA cm^−2^ with K deposition capacity of (d) 5 mA h cm^−2^ and (e) 10 mA h cm^−2^.

The a-Ti_3_C_2_ MXene frameworks with abundant macropores, large surface area, and high electrical conductivity provide ideal host materials for K deposition. Therefore, we first investigated the coulombic efficiencies of different Ti_3_C_2_ MXene based electrodes. With a plating time of 1 h at a current density of 1 mA cm^−2^ for every cycle, the coulombic efficiency of the a-Ti_3_C_2_ MXene frameworks with much improved electrochemical reaction kinetics (3.2 Ω) and Li ion diffusion coefficient (6.3 × 10^−16^ cm^2^ s^−1^) (Fig. S7 and S8, ESI†) was 99.5% for 300 cycles with a stable overpotential (60.6 mV, Fig. S9, ESI†); this result was significantly higher than those of d-Ti_3_C_2_ MXene (89.1% for 101 cycles), m-Ti_3_C_2_ MXene (81.1% for 53 cycles), Ti_3_AlC_2_ MAX (79.9% for 30 cycles) and bare Cu electrodes (9.6% for 7 cycles), as seen in Fig. 3a. When the deposition capacity was increased to 3 mA h cm^−2^ at 3 mA cm^−2^, the a-Ti_3_C_2_ MXene electrode could also maintain a high coulombic efficiency of 99.4% for 300 cycles (Fig. 3b). The superior performance of the a-Ti_3_C_2_ MXene electrode for the K anode was further proven by the voltage nucleation profiles. The d-Ti_3_C_2_ MXene, m-Ti_3_C_2_ MXene, Ti_3_AlC_2_ MAX and bare Cu electrodes had striking overpotentials of 22.3 mV, 89.0 mV, 98.6 mV and 318.2 mV in the first cycles, respectively. A flat voltage curve with a small overpotential of 19.2 mV at the nucleation stage was realized for the a-Ti_3_C_2_ MXene electrode (Fig. S10, ESI†), indicative of the exceptional potassiophilicity of the a-Ti_3_C_2_ MXene nanoribbons.^41,42^ In addition, symmetric batteries based on a-Ti_3_C_2_–K and pure K were assembled to evaluate the K plating/stripping behavior. Unlike the bare K symmetric battery, the overpotential increased sharply to 2.0 V over 20 h due to the formation of K dendrites, while the a-Ti_3_C_2_–K battery showed a flat and stable voltage hysteresis (∼14 mV) for 110 h at 3 mA cm^−2^ for 1 h (Fig. 3c and S11, ESI†). The cycling capacities were further increased to 5 mA h cm^−2^ and 10 mA h cm^−2^ at a high current density of 5 mA cm^−2^, as even the overpotential was not stable during the initial cycles due to the unstable SEI film. The plating/stripping overpotentials of the a-Ti_3_C_2_ MXene electrode still exhibited a small hysteresis of 18 mV for 800 h and 11 mV for 700 h without the short circuit, which indicates that the 3D a-Ti_3_C_2_ nanoribbon framework electrode effectively suppressed the K dendrites (Fig. 3d and e). Notably, such properties are superior to those of most reported K metal-based anodes (Table S1, ESI†), such as rGO@3D-Cu (0.5 mA cm^−2^, 0.5 mA h cm^−2^ for 200 h)^21^ and aligned carbon nanotube membrane (5 mA cm^−2^, 1 mA h cm^−2^ for 58 h).^22^

### Morphology characterization of a-Ti_3_C_2_–K hybrid anodes

**Fig. 4:**
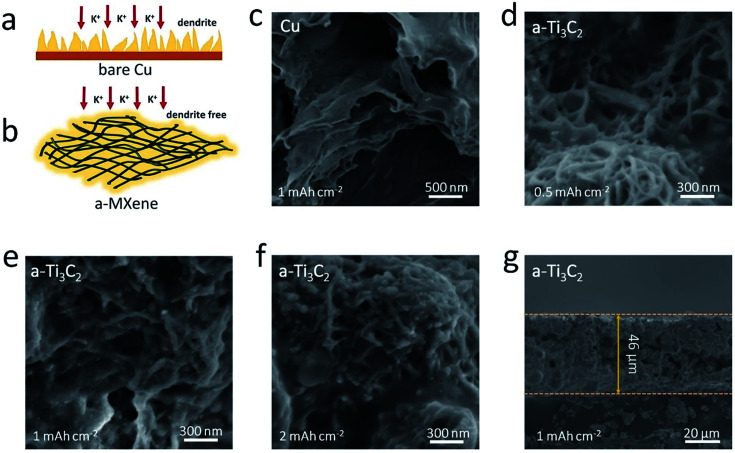
Morphology characterization of K plating on a-Ti_3_C_2_ MXene nanoribbon frameworks and bare Cu electrodes. (a and b) Schematic comparison of the different K plating behaviors on (a) bare Cu and (b) a-Ti_3_C_2_ MXene electrodes. (c) Top view SEM image of bare Cu electrode at the stage of plating K with a capacity of 1 mA h cm^−2^. (d–f) Top view SEM images of a-Ti_3_C_2_ MXene electrodes at the stage of (d) plating K with a capacity of 0.5 mA h cm^−2^, (e) 1 mA h cm^−2^, and (f) 2 mA h cm^−2^. (g) Cross section SEM image of a-Ti_3_C_2_ MXene electrode at the stage of plating K with a capacity of 1 mA h cm^−2^.

Based on the above experimental results, the 3D porous a-Ti_3_C_2_ MXene frameworks can be regarded as a prospective and available scaffold material for constructing safe and stable K metal anodes. As illustrated in Fig. 4a, if the bare Cu electrode is chosen as the matrix for K deposition, the distribution of the K nucleation sites is isolated and non-uniform due to the rough surface of Cu electrode, which result in the heterogeneous nucleation of K ions and thus the formation of detrimental K dendrites. However, when employing a-Ti_3_C_2_ MXene as the K plating scaffold, the uniformly potassiophilic functional groups with F- and O-termination on the surface coupled with the 3D porous structure, much more evenly distributed K metal nucleation sites and a smaller deposition current density are observed relative to the bare Cu electrode. Therefore, a uniform and smooth K deposition can be obtained (Fig. 4b). Scanning electron microscopy (SEM) images were used to compare the K deposition morphology. Notably, unlike for the bare Cu electrode with discernible sharp lamellar K dendrites after plating at 1 mA h cm^−2^ K metal (Fig. 4c), smooth and uniform K metal was deposited inside the 3D nanoribbon frameworks (Fig. 4d and e), even with a relatively high amount of K (2 mA h cm^−2^, Fig. 4f). A cross-section SEM image further indicated the dense and flat K metal with a thickness of ∼46 μm, and no observable K dendrites were found on the electrode (Fig. 4g).

### a-Ti_3_C_2_–K hybrid anodes for full cells

**Fig. 5:**
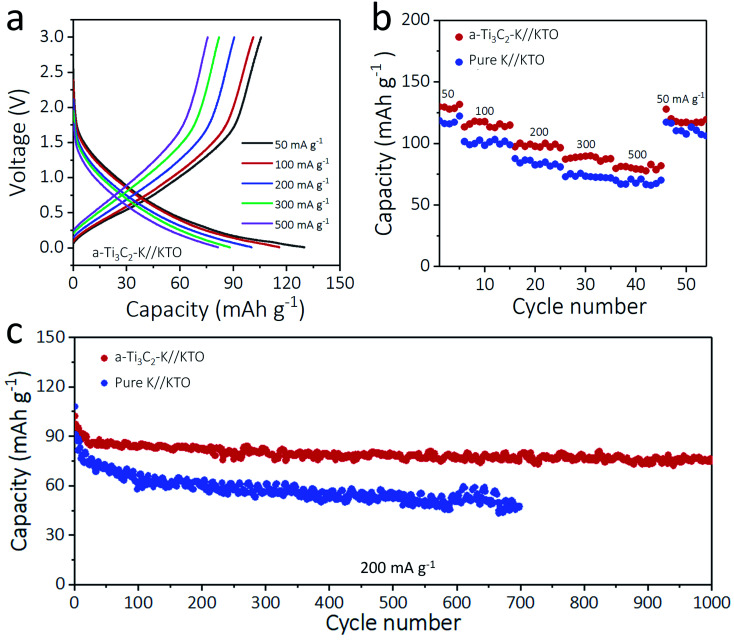
Electrochemical performance of a-Ti_3_C_2_ MXene frameworks-based K full batteries. (a) Charge and discharge profiles of a-Ti_3_C_2_–K//KTO battery tested at different rates. (b) Rate capabilities and (c) cycling stabilities of a-Ti_3_C_2_–K//KTO and pure K//KTO batteries tested at 200 mA g^−1^.

To highlight the practicality of the a-Ti_3_C_2_–K hybrid anodes, 5 mA h cm^−2^ of K was plated on a 3D a-Ti_3_C_2_ nanoribbon (a-Ti_3_C_2_–K), and a KTO cathode was obtained by alkalization and oxidation process of m-Ti_3_C_2_ MXene nanosheets at the same time.^33^ The anode and cathode were paired to assemble an a-Ti_3_C_2_–K//KTO full battery, and its electrochemical performance was compared with a pure K//KTO battery. Impressively, the a-Ti_3_C_2_–K//KTO battery exhibited superior rate performance at current densities from 50 to 500 mA g^−1^ relative to the pure K//KTO battery. For example, at 300 and 500 mA g^−1^, the a-Ti_3_C_2_–K//KTO battery showed 89.9 mA h g^−1^ and 82.9 mA h g^−1^, respectively, much higher than those of pure K//KTO battery (72.9 mA h g^−1^, 66.1 mA h g^−1^, respectively). This improved rate performance was due to the increased electrochemical kinetics and reversibility of the a-Ti_3_C_2_–K metal anode (Fig. 5a, b and S12, ESI†). The long-term cyclabilities of the a-Ti_3_C_2_–K//KTO or pure K//KTO batteries at 200 mA g^−1^ were shown in Fig. 5c. Impressively, the a-Ti_3_C_2_–K//KTO battery delivered a higher capacity of 97.5 mA h g^−1^ coupled with a lower capacity decay rate (0.023% per cycle) and high coulombic efficiency (100%) relative to that of the pure K//KTO battery (91.3 mA h g^−1^ with a capacity decay rate of 0.069% each cycle) (Fig. S13, S14 and S15 ESI†). These results further confirm the superior capability of a-Ti_3_C_2_ MXene for high-energy-density K metal batteries.

## Conclusions

In summary, we developed a 3D conductive interconnected a-Ti_3_C_2_ nanoribbon framework for dendrite free and highly stable K metal anodes. Owing to the preintercalated K in the expanded interlayer space, abundant uniformly distributed potassiophilic polar groups and 3D conductive porous framework as the host, a reduced local current density and homogenous K ion flux was realized. As a result, the nucleation and growth of K metal were well confined to the 3D skeleton. Consequently, the a-Ti_3_C_2_ MXene electrode achieved a high coulombic efficiency of 99.4% and long lifespan of 300 cycles at 3 mA cm^−2^. Impressively, a small hysteresis of 11 mV and long cycling stability (700 h) at an ultrahigh capacity of 10 mA h cm^−2^ were obtained without K dendrite growth. Furthermore, a-Ti_3_C_2_–K anodes-based a-Ti_3_C_2_–K//KTO full batteries showed a much improved rate performance and ultralow capacity decay rate of 0.023% per cycle. This rational structural engineering of K metal anodes paves the way toward an expansion of future generations of highly safe and energy-dense K metal-based batteries.

## Experimental

### Materials preparation

#### Synthesis of Ti_3_AlC_2_ MAX

The Ti_3_AlC_2_ MAX powder was prepared by a solid–liquid reaction method.^30,31^ In detail, the Ti (99%, 300 mesh), Al (99%, 10 μm) and graphite (99%, 6.5 μm) powders in a molar ratio of 3 : 1.1 : 1.88 were mixed with agate balls and absolute alcohol in an agate jar for 16 h, followed by drying at 70 °C for 8 h in air. Then, the resulting compound was uniaxially cold pressed into a green compact in a graphite mould. Subsequently, the compact was heated at 1550 °C in a furnace for 2 h in an argon flow atmosphere. Finally, the sample was naturally cooled down to room temperature.

#### Synthesis of different Ti_3_C_2_ MXene based materials

The a-Ti_3_C_2_ MXene nanoribbon frameworks were synthesized according to our previous work.^36,37^ First, m-Ti_3_C_2_ MXene nanosheets were synthesized in a sealed Teflon container by stirring treatment in atmosphere, and then the supernatant was collected.^34,35^ The a-Ti_3_C_2_ MXene nanoribbons were prepared by continuous shaking treatment (250 rpm) of m-Ti_3_C_2_ MXene (0.5 g) at room temperature (25 °C) in KOH aqueous solution (60 mL, 6 mol L^−1^) for 72 h in a sealed Teflon container under argon atmosphere, and they were harvested after rinsing and vacuum drying at 60 °C for 24 h. The Ti_3_AlC_2_ MAX powder (1.5 g) was prepared in a mixture of 30 mL aqueous HCl solution (9 mol L^−1^) with LiF (1.5 g) for 72 h at 60 °C and collected by high-speed centrifugation at 3500 rpm for 10 min. After centrifugation and washing with deionized water three times until the pH was neutral. Subsequently, the d-Ti_3_C_2_ MXene nanosheets were prepared by redispersion of the m-Ti_3_C_2_ MXene (0.1 g) in deionized water (10 mL) and sonicating for 1 h under argon.

### Materials characterization

The morphology and structure of the materials were characterized by SEM (JEOL JSM-7800F), TEM (JEM-2100), XPS (Thermo ESCALAB 250Xi equipped with monochromatic Al Kα source of 1486.5 eV), and XRD (Empyrean with Cu Kα radiation in the 2*θ* range from 5 to 90°), Nitrogen adsorption and desorption isotherms were carried out to investigate the specific surface area and pore size distribution of the products.

### Electrochemical measurement

The as-obtained Ti_3_AlC_2_ MAX, m-Ti_3_C_2_ MXene, d-Ti_3_C_2_ MXene or a-Ti_3_C_2_ MXene as active materials and polyvinylidene fluoride as binder (mass ratio of active materials: binder = 9 : 1) were mixed into a slurry by stirring in *N*-methyl-2-pyrrolidone (NMP) for 24 h. Then, the slurry was coated onto Cu foil and dried in a vacuum drying oven at 100 °C for 12 h. Further, the foil was punched into disks as the working electrode. The loading mass of materials was approximately ∼1 mg cm^−2^, and bare Cu foil was also punched for reference. All the batteries were assembled with standard CR2016 coin-type batteries in an argon-filled glovebox with O_2_ and H_2_O content below 0.5 ppm. The electrolyte was 0.8 M potassium bis(fluorosulfonyl)imide (KFSI) in ethylene carbonate (EC): diethyl carbonate (DEC) (1 : 1, v/v) and one piece of glass fiber (GF/A, Whatman) was used as the separator. About 100 μL electrolyte was dropped into each battery. Ti_3_AlC_2_ MAX, m-Ti_3_C_2_ MXene, d-Ti_3_C_2_ MXene, a-Ti_3_C_2_ MXene or bare Cu electrodes were severed as the working electrodes, and K metal was used as the counter/reference electrodes to evaluate the coulombic efficiency. The assembled batteries were precycled between 0.01 and 3 V at 0.1 mA 5 times to stabilize the SEI formation and remove surface contamination. Afterwards, a certain capacity of K was deposited onto the electrodes and then charged to 1 V (*vs.* K^+^/K) to strip the K at a certain current density for each cycle on a LAND CT2001A battery system. Symmetric battery configurations were assembled with a-Ti_3_C_2_–K or pure K anodes to evaluate the long-time cycling stability. For the full battery test, the KTO was prepared by a simultaneous oxidation and alkalization process of Ti_3_C_2_ MXene as we previously reported.^33^ The well-mixed slurry containing 80 wt% of KTO, 10 wt% of carbon black, and 10 wt% of polyvinylidene fluoride with NMP solvent was cast onto carbon-coated Al foil using the doctor blade technique and then dried under vacuum at 120 °C for 12 h. The areal loading of the KTO was about 1 mg cm^−2^. The a-Ti_3_C_2_–K and pure K anodes were obtained from preprocessed half batteries. After depositing a certain amount of K metal (5 mA h cm^−2^) onto the current collector, the battery was disassembled in the glove box, and the dismantled K anodes were further reassembled into a full battery against a KTO cathode, using 0.8 M KPF_6_ in EC/DEC (v/v = 1 : 1) as the electrolyte. Glass fiber (GF/A, Whatman) was used as a separator and around 100 μL electrolyte was dropped in each cell. The CV curves were obtained on a CHI 760E electrochemical workstation at 0.1 mV s^−1^. The EIS measurements were carried out using a CHI 760E electrochemical workstation by applying an ac amplitude of 5 mV over the frequency range of 100 kHz to 0.01 Hz.

## Conflicts of interest

There are no conflicts to declare.

## Supplementary Material

NA-002-D0NA00515K-s001
