# Supplementary material for: Three dimensional Ti3C2 MXene nanoribbon frameworks with uniform potassiophilic sites for the dendrite-free potassium metal anodes
Source: Nanoscale Adv. 2020 Jul 27;2(9):4212–9. doi: 10.1039/d0na00515k (PMC9417470; doi:10.1039/d0na00515k)
Supplement: NA-002-D0NA00515K-s001 [file NA-002-D0NA00515K-s001.pdf]

Electronic Supplementary Material (ESI) for Nanoscale Advances.
This journal is © The Royal Society of Chemistry 2020

Electronic Supplementary Material for Nanoscale Advances.

This journal is © The Royal Society of Chemistry 2020

Supplemental Information

Three Dimensional Ti_3C_2 MXene Nanoribbons Frameworks with Uniform Potassiophilic Sites for Dendrite-Free Potassium Metal Anodes

Haodong Shi,[abc] Yanfeng Dong,[d] Shuanghao Zheng,[ac] Cong Dong,[abc] Zhong-Shuai Wu*[ac]

Affiliations:

[a]State Key Laboratory of Catalysis, Dalian Institute of Chemical Physics, Chinese Academy of Sciences, 457 Zhongshan Road, Dalian 116023, China

[b]University of Chinese Academy of Sciences, 19 A Yuquan Rd, Shijingshan District, Beijing 100049, China

[c]Dalian National Laboratory for Clean Energy, Dalian Institute of Chemical Physics, Chinese Academy of Sciences, 457 Zhongshan Road, Dalian 116023, China

[d]Department of Chemistry, College of Sciences, Northeastern University, 3-11 Wenhua Road, Shenyang 110819, China

*Corresponding author: wuzs@dicp.ac.cn

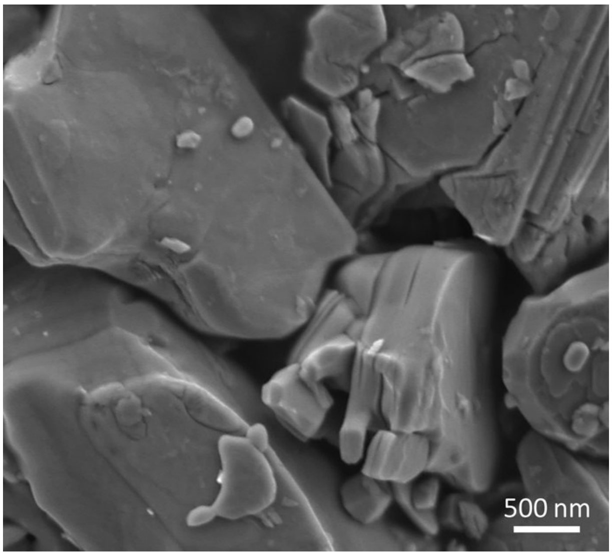

Fig. S1 SEM image of Ti_3AlC_2 MAX.

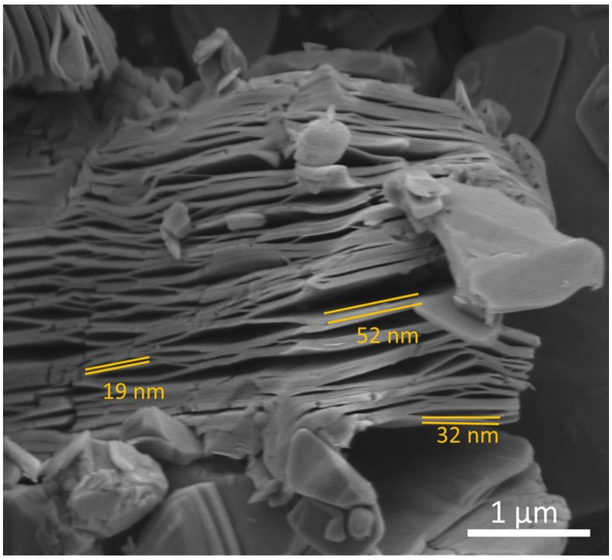

Fig. S2 SEM image of m-Ti_3C_2 MXene nanosheets.

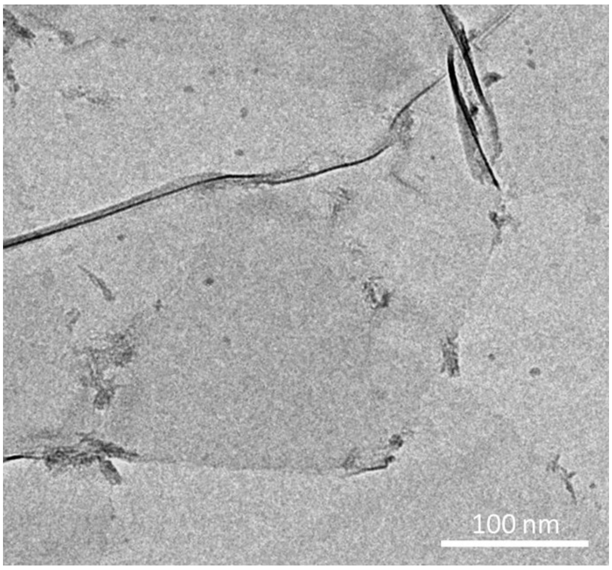

Fig. S3 TEM image of d-Ti_3C_2 MXene nanosheets.

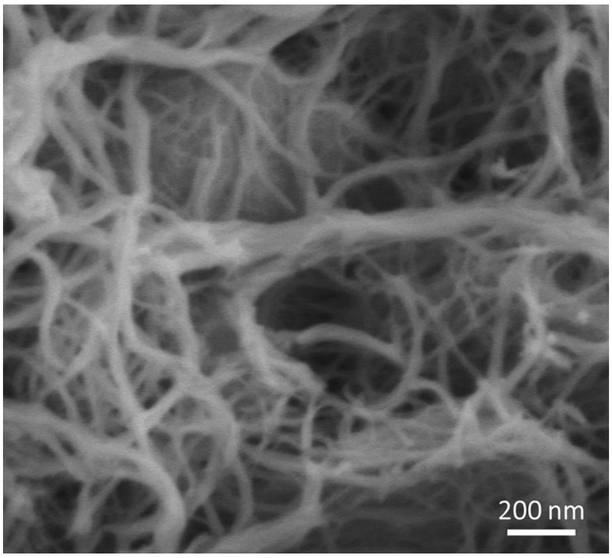

Fig. S4 SEM image of a-Ti_3C_2 MXene nanoribbon frameworks.

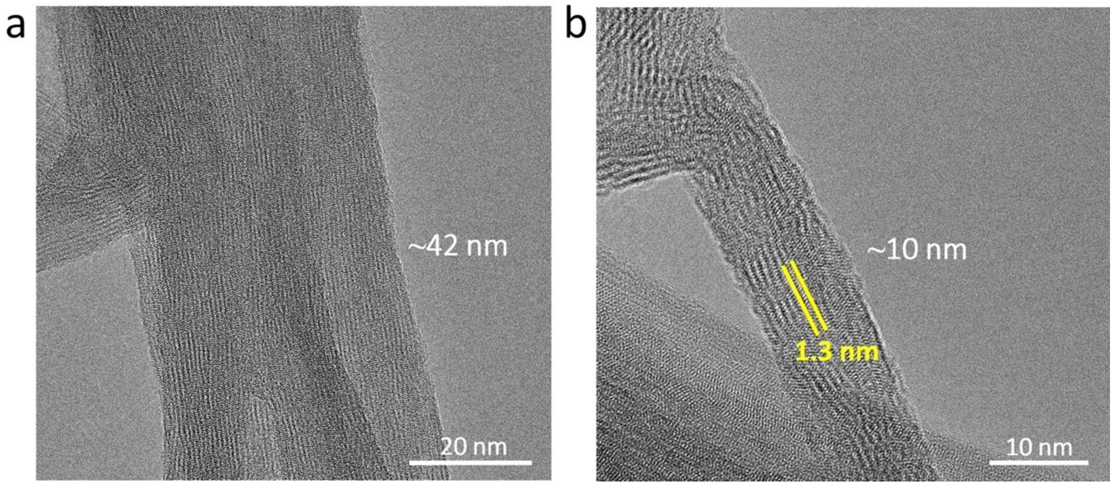

Fig. S5 (a,b) HRTEM images of a-Ti_3C_2 MXene nanoribbons.

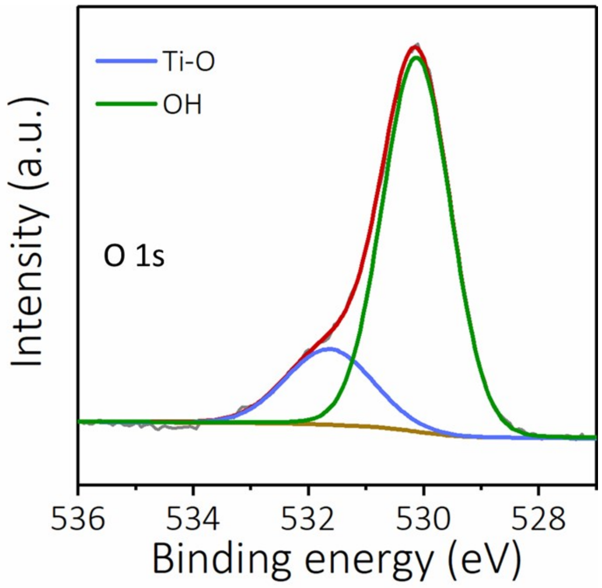

Fig. S6 High resolution O1s XPS spectrum of a-Ti_3C_2 nanoribbons.

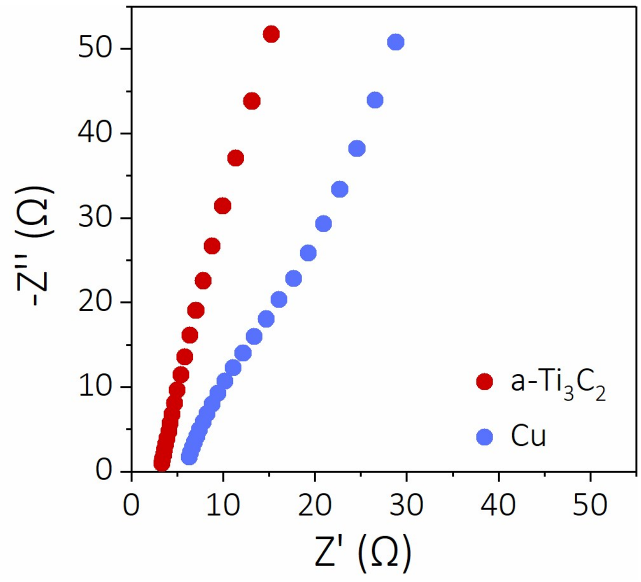

Fig. S7 Nyquist plots of the a-Ti_3C_2 MXene/K and bare Cu/K asymmetric batteries.

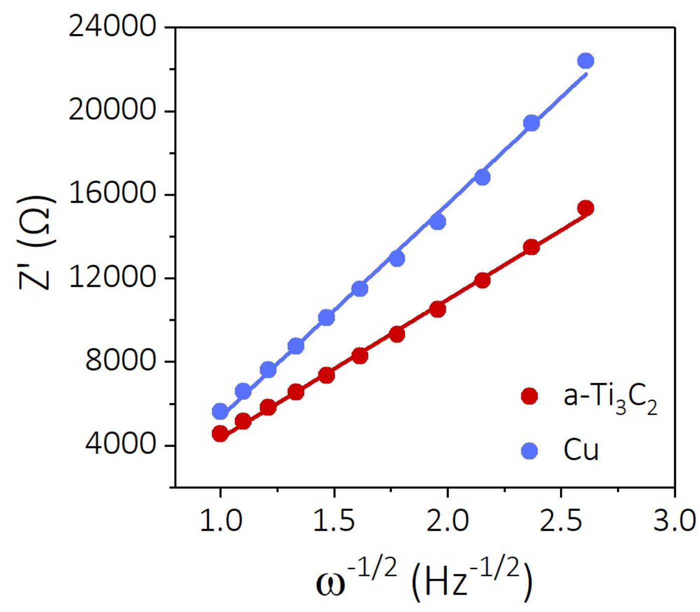

Fig. S8 The relationship of between Z' and square root of the frequency ($\omega^{-1/2}$) in the low-frequency region for the a-Ti_3C_2 MXene/K and bare Cu/K asymmetric batteries.

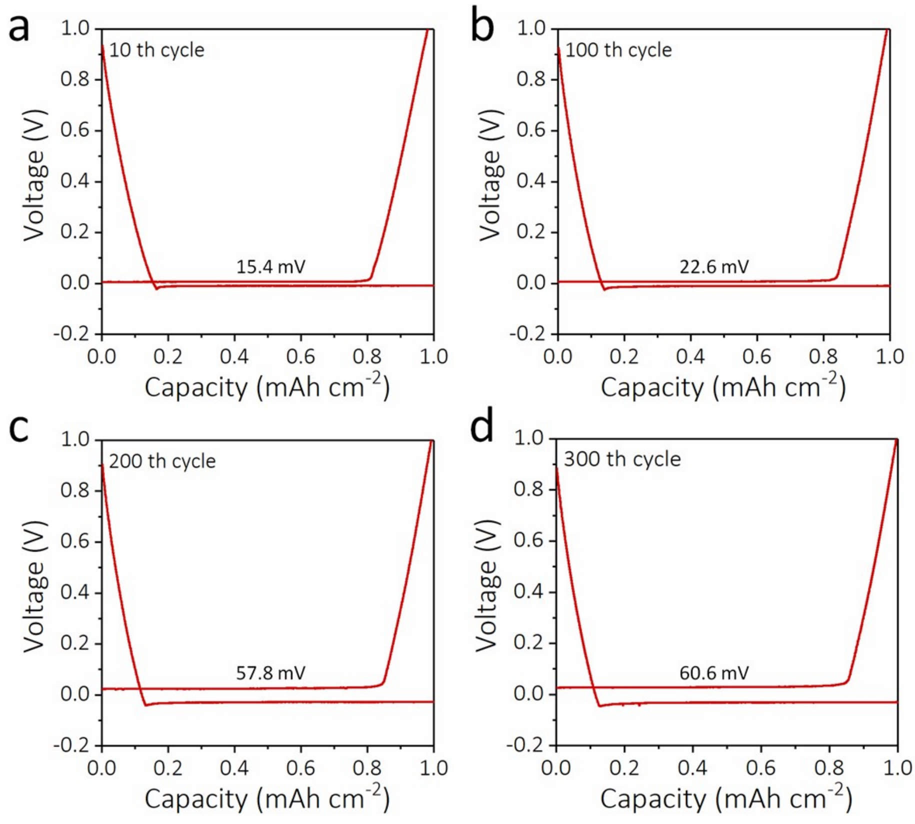

Fig. S9 Voltage profiles of a-Ti_3C_2 MXene electrode tested at different cycles. Plant 1 mAh cm^{-2} of K at a current density 1 mA cm^{-2} at (a) 10th cycle, (b) 100th cycle, (c) 200th cycle, and (d) 300th cycle.

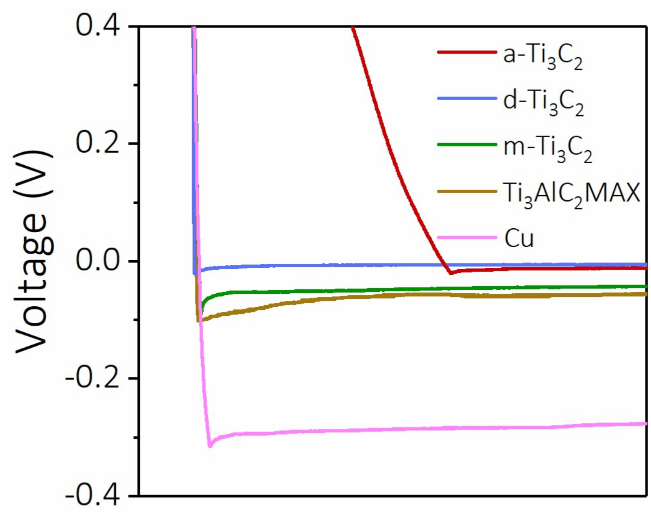

Fig. S10 Nucleation section on the voltage profiles of a-Ti_3C_2 MXene, d-Ti_3C_2 MXene, m-Ti_3C_2 MXene, Ti_3AlC_2 MAX and bare Cu electrodes with K deposition capacity of 1 mAh cm^{-2} at 1 mA cm^{-2}.

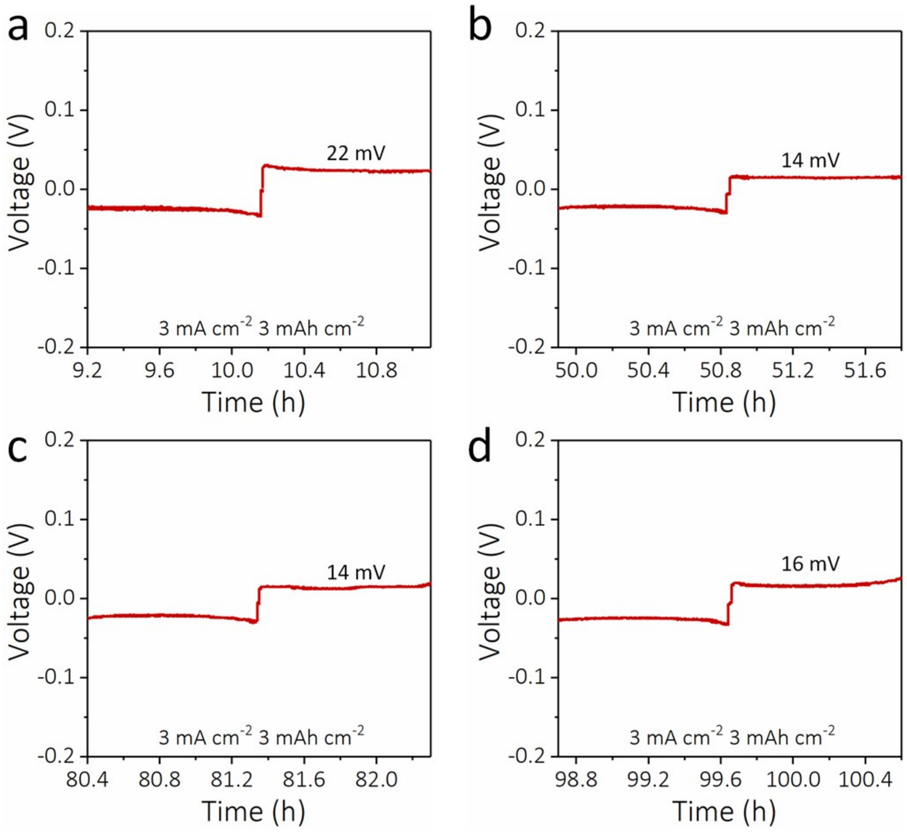

Fig. S11 Detailed voltage profiles of a-Ti_3C_2-K symmetric battery at different time. Planting 3 mAh cm^{-2} of K at current density of 3 mA cm^{-2} for (a) 10 h, (b) 50 h, (c) 80 h, and (d) 100 h, respectively.

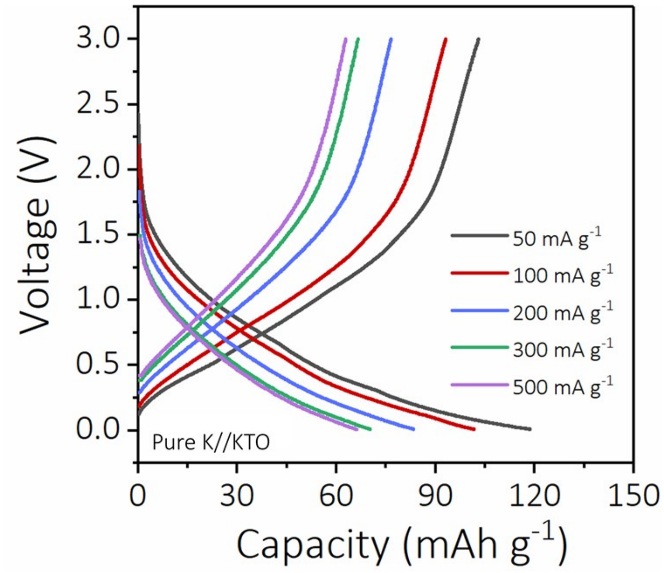

Fig. S12 Charge and discharge profiles of pure K//KTO battery tested at different rates.

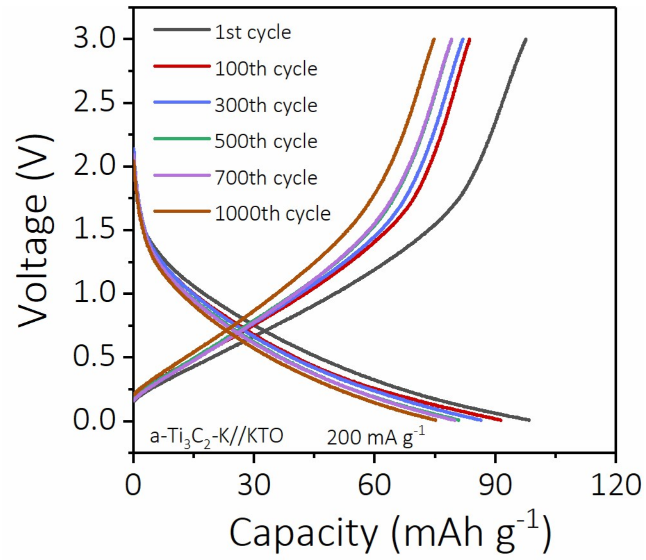

Fig. S13 Charge and discharge profiles of a-Ti_3C_2-K//KTO battery obtained at different cycles at 200 mA g^{-1}.

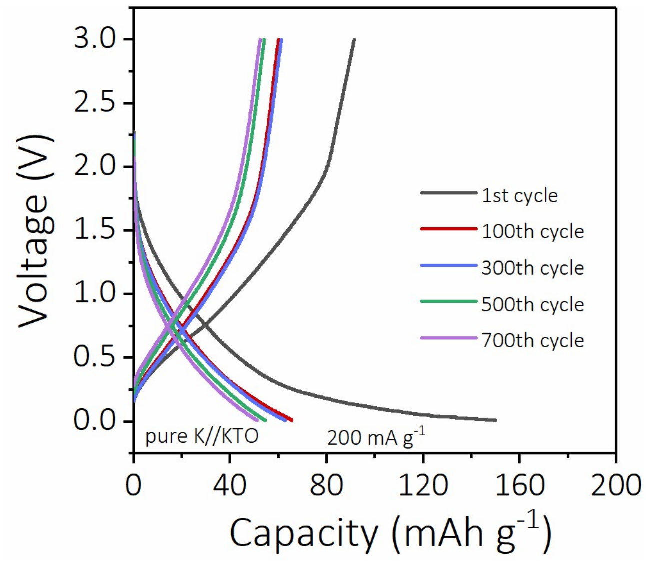

Fig. S14 Charge and discharge profiles of pure K//KTO battery obtained at different cycles at 200 mA g^{-1}.

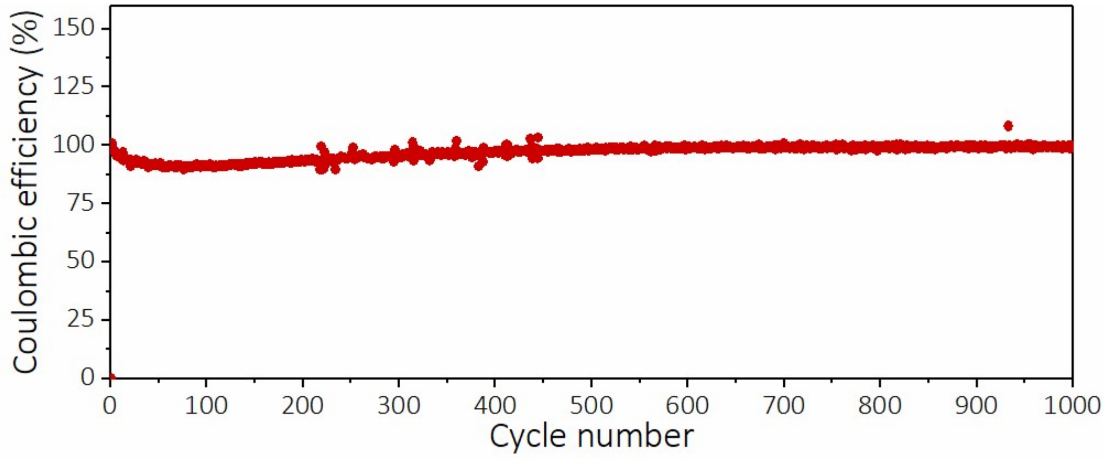

Fig. S15 Coulombic efficiency of a-Ti_3C_2-K//KTO battery tested at 200 mA g^{-1}.

Table S1. Performance comparison of a-Ti_3C_2-K electrode with the state-of-the-art K anodes recently reported

**Anode materials**	**Current density ($mA\ cm^{-2}$)**	**Areal capacity ($mAh\ cm^{-2}$)**	**Cycle time (h)**	**Refs.**
rGO@3D-Cu	0.5	0.5	200	Ref. [1]
ACM	5	1	58	Ref. [2]
PCNF@SnO_2	1	10	350	Ref. [3]
DN-MXene/CNT	0.5	0.5	300	Ref. [4]
HNCP/G	1	1	100	Ref. [5]
PM/NiO	0.4	0.1	100	Ref. [6]
K-Hg	0.5	0.5	250	Ref. [7]
Polished K metal	0.1	0.02	80	Ref. [8]
**a-Ti_3C_2**	**5**	**10**	**700**	**This work**
	**5**	**5**	**800**	

ACM: ligned carbon nanotube membrane; PCNF@SnO_2: SnO_2-coated conductive porous carbon nanofiber framework; DN-MXene: defect-rich and nitrogen-containing MXene; HNCP/G: hollow N-doped C polyhedrons/graphene; PM: puffed millet.

References

1. P. Liu, Y. Wang, Q. Gu, J. Nanda, J. Watt and D. Mitlin, *Adv. Mater.*, 2020, **32**, 1906735.
2. L. Qin, Y. Lei, H. Wang, J. Dong, Y. Wu, D. Zhai, F. Kang, Y. Tao and Q.-H. Yang, *Adv. Energy Mater.*, 2019, **9**, 1901427.
3. X. Zhao, F. Chen, J. Liu, M. Cheng, H. Su, J. Liu and Y. Xu, *J. Mater. Chem. A*, 2020, **8**, 5671-5678.
4. X. Tang, D. Zhou, P. Li, X. Guo, B. Sun, H. Liu, K. Yan, Y. Gogotsi and G. Wang, *Adv. Mater.*, 2020, **32**, 1906739.
5. M. Ye, J.-Y. Hwang and Y.-K. Sun, *ACS Nano*, 2019, **13**, 9306-9314.
6. Y. Li, L. Zhang, S. Liu, X. Wang, D. Xie, X. Xia, C. Gu and J. Tu, *Nano Energy*, 2019, **62**, 367-375.
7. Q. Yang, Y. Ding and G. He, *Chem. Commun.*, 2020, **56**, 3512-3515.
8. Y. Gu, W.-W. Wang, Y.-J. Li, Q.-H. Wu, S. Tang, J.-W. Yan, M.-S. Zheng, D.-Y. Wu, C.-H. Fan, W.-Q. Hu, Z.-B. Chen, Y. Fang, Q.-H. Zhang, Q.-F. Dong and B.-W. Mao, *Nat. Commun.*, 2018, **9**, 1339.
